# Antinociceptive effect of lidocaine, tramadol, and their combination for lumbosacral epidural analgesia in rabbits undergoing experimental knee surgery

**DOI:** 10.1186/s12917-022-03360-y

**Published:** 2022-06-29

**Authors:** Mohamed Salem, Awad Rizk, Esam Mosbah, Adel Zaghloul, Gamal Karrouf, Marwa Abass

**Affiliations:** Department of Surgery, Anesthesiology, and Radiology, Faculty of Veterinary Medicine, Mansoura University, Mansoura, 35516 Egypt

**Keywords:** Lumbosacral, Lidocaine, Tramadol, Ketamine, Rabbits

## Abstract

**Aim:**

The current study aimed to evaluate the antinociceptive effect of lidocaine, tramadol, and their combination for lumbosacral epidural analgesia in rabbits undergoing knee surgery.

**Materials and methods:**

This study was performed on 24 male New Zealand white rabbits weighing 2.8 to 3.0 kg and was allocated into three groups. All groups were anaesthetized by intramuscular (IM) injection of 35 mg/kg ketamine and 5 mg/kg xylazine, 0.1 mg/kg butorphanol. Rabbits in Group A received epidural analgesia of 4 mg/kg lidocaine 2%; Group B rabbits received epidural analgesia of 4 mg/kg tramadol 5%, and Group C rabbits received epidural analgesia of a combination of 4 mg/kg lidocaine and 4 mg/kg tramadol. Prior to and during surgery, the following parameters were recorded in a regular pre-set time interval: onset time of analgesia (OT), duration of flaccid paralysis (DFP), duration of analgesia (DA), onset and duration of sensory blockade, onset and duration of motor blockade, heart rate (HR), respiratory rate (RR), and rectal temperature (RT).

**Results:**

The mean OT demonstrated a significant decrease (*P* < 0.05) in Group C (46.5 ± 1.4 sec) compared to Group A and B (61.0 ± 2.4 and 54.5 ± 3.5 sec), respectively. DFP was significantly lower (*P* < 0.05) in Group C (35.5 ± 2.9 min) than in Group A and B (17.6 ± 1.4 and 21.8 ± 3.6), respectively. DA showed a significant increase (*P* < 0.05) in group C (45.8 ± 3.3 min) compared to groups A and B, respectively (23.3 ± 1.1 and 31.5 ± 2.3). Heart rate, RR, and RT significantly decreased in Group C compared to the other groups.

**Conclusion:**

According to the current study findings, lumbosacral epidural administration of lidocaine combined with tramadol could be a better choice for potentiating the analgesia than administration of either drug separately and may be safely used in rabbits undergoing knee surgery.

## Background

Due to its proximity to the spinal cord receptors responsible for the modulation and transmission of nociceptive signals, lumbosacral analgesia is an effective method of analgesia [1, 2]. Lidocaine HCl is the most commonly used anesthetic agent administrated for epidural analgesia that is characterized by a rapid onset of action and effective desensitization [3]. Since the duration of analgesia after lidocaine is relatively short, re-dosing of the agent is required for relatively long surgical operations [4]. Combining local anesthetic with tranquilizers or opioids results in a more extended duration of analgesia and decreases the dose and the side effects of the anesthetic drugs [5–7].

Opioids are used to improve the quality, extend the duration of analgesia, speed up the onset time of analgesia, and prolong the duration of flaccid paralysis and analgesia [8]. Lumbosacral use of opioids seems to be a valuable tool for managing postoperative pain, mainly as part of a balanced analgesia protocol [9]. Opioids are used to improve analgesia as an adjuvant to local anesthetic [10] and ketamine [11, 12]. Tramadol is a synthetic analogue of codeine that has been demonstrated to provide prolonged epidural analgesia in humans [13]. Tramadol utilization has been reported for the management of moderate, severe pain and postoperative epidural analgesia in cattle [14], goats [15], dogs [16], horses [17], camels [10], rabbits [18], and cats [19].

Rabbits are commonly used in experimental orthopedic research, where potent analgesia is required [20]. However, the use of xylazine and ketamine anesthesia in rabbits is commonly used in experimental research, particularly in orthopedic ones, because it provides adequate anesthesia with no pain response. The nociception may still not be completely alleviated. The current research aimed to investigate the use of lumbosacral administration of lidocaine and tramadol to improve the analgesic effect in rabbits undergoing knee surgery.

## Results

The mean OT was significantly decreased (*P* < 0.05) in Group C (46.5 ± 1.4 seconds) compared to groups A and B, with values of (61.0 ± 2.4 and 54.5 ± 3.5 seconds), respectively. The mean DFP was significantly increased (*P* < 0.05) in Group C (35.5 ± 2.9 minutes) compared to groups Group A and B, with means of (17.6 ± 1.4 and 21.8 ± 3.6 minutes) respectively. The mean DA was significantly increased (*P* < 0.05) in Group C (45.8 ± 3.3 minutes) compared to Group A and B (23.3 ± 1.1 and 31.5 ± 2.3 minutes), respectively. Mean sensory blockade onset was significantly decreased in Group C (1.0 ± 0.1 minutes) compared to groups A and B, with values of (1.5 ± 0.4 and 1.6 ± 0.3 minutes) respectively. The mean time to the maximum sensory blockade was significantly decreased in Group C (3.1 ± 0.3 minutes) compared to groups A and B (3.6 ± 0.6 and 3.7 ± 0.3 minutes), respectively. The mean duration of sensory blockade was significantly increased (*P* < 0.05) in group C (57.8 ± 1.7 minutes) compared to groups A and B, with values of (54.2 ± 1.2 and 54.1 ± 1.2 minutes), respectively (Table 1).

**Table 1:** The mean ± standard deviation of onset time (OT) (sec), duration of flaccid paresis (DFP) (min), duration of analgesia (DA) (min), sensory blockade onset (min), time to maximum sensory blockade (min), duration of sensory blockade (min), motor blockade onset (min), and time to maximum motor blockade (min) in rabbits. Group A: lidocaine epidural, Group B: tramadol epidural, and Group C: lidocaine- tramadol epidural

Parameters	Group A (Lidocaine)	Group B (Tramadol)	Group C (Lidocaine and Tramadol)
Onset time (OT) (sec)	62.8 ± 3.3 ^a^	54.5 ± 3.5 ^b,*^	46.5 ± 1.4 ^c,*^
Duration of flaccid paresis (DFP) (min)	17.6 ± 1.4 ^a^	21.8 ± 3.6 ^b,*^	35.5 ± 2.9 ^c,*^
Duration of analgesia (DA) (min)	23.3 ± 1.1 ^a^	31.5 ± 2.3 ^b,*^	45.8 ± 3.3 ^c,*^
Sensory blockade onset (min)	1.5 ± 0.4 ^a^	1.6 ± 0.3 ^a^	1.0 ± 0.1 ^b,*^
Time to maximum sensory blockade (min)	3.6 ± 0.6 ^a^	3.7 ± 0.3 ^a^	3.1 ± 0.3 ^a^
Duration of sensory blockade (min)	54.2 ± 1.2 ^a^	54.1 ± 1.2 ^a^	57.8 ± 1.7 ^b,*^
Motor blockade onset (min)	1.8 ± 0.3 ^a^	1.6 ± 0.2 ^a^	1.1 ± 0.1 ^b,*^
Time to maximum motor blockade (min)	3.5 ± 0.4 ^a^	3.5 ± 0.5 ^a^	2.6 ± 0.2 ^b,*^
Duration of motor blockade (min)	31.7 ± 1.6 ^a^	30.5 ± 2.9 ^a^	37.0 ± 1.8 ^b,*^

Means with different superscript litters are significantly different at *P* < 0.05

All within interaction, Wilks’ Lambda, *P* < 0.0001; Within time, Wilks’ Lambda, *P* < 0.0001; Time * groups, Wilks’ Lambda, *P* < 0.0001

The mean motor blockage onset time was significantly decreased in Group C to reach (1.1 ± 0.1 minutes) compared to groups A and B, which have (1.8 ± 0.3 and 1.6 ± 0.3 minutes) respectively. Group C had a significantly shorter mean time to maximum motor blockade (2.60.2 minutes) than groups A and B, which had (3.5 ± 0.4 and 3.5 ± 0.5 minutes) respectively. The mean duration of motor blockade was significantly increased (*P* < 0.05) in group C (37.0 ± 1.8 minutes) compared to groups A and B, with values of (31.7 ± 1.6 and 30.5 ± 2.9 minutes) respectively.

The mean HR was significantly decreased in all groups (*P* < 0.05) from baseline, with the lowest value recorded at 60 min postoperatively with the following values: (124.0 ± 0.6 vs. 144 ± 0.4 in Group A, 123.7 ± 0.8 vs. 144 ± 0.4 in Group B, and 103.7 ± 0.5 vs. 145 ± 1.5 beat per min in Group C). The mean RR was significantly decreased in groups A and B (*P* < 0.05) from baseline (60 ± 0.6), with the lowest value recorded at 60 min postoperatively to be 49.7 ± 0.7 and 44.3 ± 0.8 breaths per min, respectively. The mean RR was significantly decreased in group C (*P* > 0.05) from baseline (59.5 ± 0.8), with the lowest value recorded at 75 min postoperatively (34.3 ± 0.8 breaths per min). The mean RT was significantly decreased in all groups (*P* < 0.05), with the lowest value recorded at 60 min postoperatively (38.3 ± 0.05 vs. 39.4 ± 0.08 C^°^ in Group A, 37.2 ± 0.04 vs. 39.5 ± 0.07 C^°^ in Group B, and 37.2 ± 0.04 vs. 39.5 ± 0.07 C^°^ in Group C; Table 2).

**Table 2:** The mean ± standard deviation of HR (beat/ min), RR (breath/ min) and RT (°C) measured at different times 10 minutes prior- and 10, 20, 30, 45, 60, 75, and 90 minutes post- the epidural analgesia in rabbits. Group A: lidocaine epidural, group B: tramadol epidural, and group C: lidocaine-tramadol epidural

Variables	Groups	Monitoring time (minutes)							
		−10	10	20	30	45	60	75	90
**HR (beat/min)**	**A**	144.0 ± 0.4 ^a^	142.0 ± 0.5 ^a^	134.6 ± 0.5 ^b^	132.0 ± 0.6 ^b^	124.5 ± 0.8 ^b^	124.0 ± 0.6 ^*b^	127.5 ± 0.8 ^*b^	137.5 ± 0.8 ^b^
	**B**	144.0 ± 0.4 ^a^	143.0 ± 0.6 ^a^	140.8 ± 0.8 ^a^	131.0 ± 0.8 ^a^	127.7 ± 0.5 ^a^	123.0 ± 0.8 ^*b^	127.3 ± 0.8 ^*b^	135.5 ± 0.8 ^a^
	**C**	145.0 ± 1.5 ^a^	128.0 ± 0.9 ^*b^	115.3 ± 0.8 ^*c^	108.0 ± 1.1 ^*b^	107.0 ± 1.4 ^c^	103.7 ± 0.5 ^*c^	122.7 ± 0.8 ^*b^	131.3 ± 0.8 ^c^
**RR (breath/min)**	**A**	60.0 ± 0.6 ^a^	55.7 ± 0.8 ^a^	51.5 ± 0.5 ^a^	48.2 ± 0.7 ^a^	47.3 ± 0.8 ^a^	49.7 ± 0.7 ^*b^	53.2 ± 0.8 ^*b^	59.7 ± 1.0 ^a^
	**B**	60.2 ± 0.9 ^a^	57.0 ± 0.6^a^	54.3 ± 0.8 ^*b^	45.8 ± 0.7 ^*b^	44.3 ± 0.8 ^a,b^	44.3 ± 0.8 ^*c^	48.0 ± 0.6 ^*c^	59.8 ± 1.4 ^a^
	**C**	59.5 ± 0.8 ^a^	52.0 ± 1.9 ^*b^	46.0 ± 0.9 ^*c^	43.3 ± 0.8 ^*c^	41.0 ± 0.9 ^b^	37.0 ± 0.6 ^*d^	34.3 ± 0.8 ^*d^	43.3 ± 0.8 ^*b^
**RT °C**	**A**	39.4 ± 0.08 ^a^	39.3 ± 0.05 ^a^	39.2 ± 0.09 ^a^	38.4 ± 0.08 ^* b^	38.4 ± 0.06 ^b^	38.3 ± 0.05 ^*b^	39.0 ± 0.05 ^a,b^	39.2 ± 0.05 ^a, b^
	**B**	39.5 ± 0.07 ^a^	39.4 ± 0.05 ^b^	39.2 ± 0.05 ^a^	38.6 ± 0.08 ^b^	38.4 ± 0.05 ^b^	38.3 ± 0.05 ^b^	39.0 ± 0.05 ^b^	39.3 ± 0.04 ^b^
	**C**	39.5 ± 0.07 ^a^	39.1 ± 0.05 ^b^	38.2 ± 0.06 ^b^	37.5 ± 0.06 ^*c^	37.2 ± 0.06 ^c^	37.2 ± 0.04 ^*c^	37.7 ± 0.08 ^*c^	38.8 ± 0.06 ^b^

Means with different superscript letters are significantly different at *P* < 0.05 among groups

Means with an asterisk (*) differ significantly (*P* < 0.05) from baseline

All within interaction, Wilks’ Lambda, *P* < 0.001; Within time, Wilks’ Lambda, *P* < 0.0001; Time * groups, Wilks’ Lambda, *P* < 0.001

## Discussion

Although xylazine–ketamine anesthesia causes a state of unconsciousness and the animals do not experience pain, where the nociception may not be abolished entirely [3, 21]. While the clinical evaluation of adequate intraoperative antinociception may be difficult, increased HR, RR, and RT may be observed in anesthetized animals in response to extensive surgical stimulation [5]. Meanwhile, blocking these responses by increasing xylazine and ketamine doses could result in severe cardio-respiratory depression [3]. Consequently, the present study aimed to evaluate the efficacy of lidocaine, tramadol, and their combination in producing intraoperative antinociception and analgesia in the immediate postoperative period without inducing clinically significant changes in HR, RR, and RT.

Rabbits were used in the present study because many reports have revealed that rabbits are a good model for studying the epidural block technique and evaluating the sensory and motor loss under standardized experimental conditions [22, 23]. Epidural analgesia could be used in painful surgical procedures for intraoperative and postoperative analgesia as well as continuous pain relief, particularly in animals with chronic pain [18].

Induction of lumbosacral epidural analgesia in ferrets and rabbits is bearly identical to that of the technique described for dogs and cats, with the exception that at the time of entry into the epidural space, the definitive popping sensation is rarely detected when the inner arcuate ligament is punctured [18]. Tramadol has been used intravenously, resulting in prolonged analgesia without severe side effects in horses [24]. Herein, the mean onset time of analgesia was significantly shorter with lidocaine tramadol combination than in the other groups (*P* < 0.05). Moreover, the duration of analgesia was longer with the lidocaine tramadol combination than in the other groups (*P* < 0.05), indicating that adding tramadol to lidocaine accelerates and prolongs the onset of analgesia. Similar findings were reported in a previous study conducted on ruminants and donkeys [25, 26].

The mean duration of flaccid paralysis was significantly longer in the lidocaine tramadol combination group compared to the other groups (*p* < 0.05). This finding can be attributed to a synergistic effect between lidocaine and tramadol. Combining the two drugs can decrease the side effects of each individual drug and increase the duration of flaccid paralysis and analgesia [27].

Tramadol is an analgesic with central effects acting on opioid receptors and inhibiting the reuptake of norepinephrine and serotonin [13]. The mean duration of sensory and motor blockade was longer in the lidocaine tramadol combination group compared to the other groups (*p* < 0.05), which was also reported in Tavakoli’s study [18]. In groups A, B, and C, the mean HR, RR, and RT decreased significantly from baseline. In contrast to Atiba’s research [28], who reported that HR, RR, and RT were non-significance different from baseline values after epidural administration of lidocaine and tramadol alone or in combination in buffalo calves.

## Conclusion

In conclusion, the findings of this study indicated that the combination of tramadol-lidocaine produced good analgesia with relatively rapid onset and increased duration of action compared to each drug alone. This combination may also be beneficial in the clinical practice for a longer duration in orthopedic surgery.

## Materials and methods

### Study sample

In this study, a total of 24 male New Zealand white rabbits weighing 2.8 to 3.0 kg were used in this study. The rabbits were kept in the animal house of Mansoura Veterinary Teaching Hospital, Faculty of Veterinary Medicine, Mansoura University, Egypt, for two weeks before starting the experiment. All animals had free access to food and water and were kept under standardized conditions. Animals were allowed to acclimatize to their cages and surroundings for several days. This study was approved by the Scientific Research Ethical Committee, Faculty of Veterinary Medicine, Mansoura University, Egypt Code No. Ph.D./15.

### Study design

All rabbits received an intramuscular injection of xylazine HCl (Xylaject, 20 mg/ ml, ADWIA, Cairo, Egypt) at a dose of 5 mg/kg and ketamine HCl (Aneket®, 50 mg/ ml, NEON Laboratories Ltd., Mumbai, India) at the dose of 15 mg/kg. The technique of lumbosacral injection in rabbits is similar to that described in dogs [29]. The lumbosacral area was aseptically prepared. The hind limbs of the rabbits were flexed to allow the greatest opening of the lumbosacral space. The epidural injections were performed via 50 mm, 20-gauge needles. Before the injection, aspiration was used to ensure that the needle did not penetrate the blood vessels. The correct placement of the needle was confirmed by the hanging drop technique and lack of resistance during the injection. The rabbits were randomly allocated into three groups (*n* = 8) as follows:

Group A: lumbosacral administration of 4 mg/kg of lidocaine (Debocaine, 20 mg/ ml, the Arab Company for Gelatin and Pharmaceutical industries, Cairo, Egypt).

Group B: lumbosacral administration of 4 mg/kg tramadol (Minpharm, 50 mg/ mL, Grünethal, Germany).

Group C: lumbosacral administration of a combination of 4 mg/kg lidocaine HCL and 4 mg/kg tramadol.

### Study design

The knee joint was aseptically prepared, and an anteromedial parapatellar incision was used to open the joint. Using an electric drill with a 3.5 mm diameter bit, an osteochondral defect of 5 mm depth and 4 mm width was created in the middle of the trochlear groove. The joint was flushed with sterile normal saline. The joint capsule was sutured using a 3–0 polyglycolic acid suture (EGYSORB, Taisier Med, Cairo, Egypt) with a simple continuous suture pattern. The subcutaneous tissues were closed using a subcuticular suture pattern using the same suture material, and the skin incision was closed using polypropylene 2\0 (PROLENE, ETHICON, USA). In all groups, the mean average duration of surgery was 15 ± 1.1 min.

### Monitoring

The heart rate (HR) (beats/min) was recorded by auscultation using a stethoscope, respiratory rate (RR) (breath/ min) was documented by counting thoracic excursions, and the rectal temperature (RT) was documented using a digital temperature. These parameters were evaluated at − 10, 10, 20, 30, 45, 60, 75, and 90 minutes before and after lumbosacral drug administration.

The sensory blockade was recorded by observing an aversive reaction to pinprick stimulus with a needle (18-gauge) starting from the sacral to thoracic skin every minute for 5 minutes after lumbosacral injection and then every five minutes till the sensory blockade vanished. The motor activities were continuously recorded and assessed every 30 sec till reaching the motor blockade peak intensity and then evaluated every five minutes.

The lumbosacral epidural anesthetic indices were recorded for each rabbit according to [5, 18], which include:

Time to onset of analgesia (OT): Time that elapsed between injection of the drug and loss of response to pinprick.

Duration of flaccid paralysis (DFP): Time that elapsed between injecting the drug and rabbits becoming ambulatory.

Duration of analgesia (DA): Time that elapsed between injection of the drug and return of response to pinprick in the tail and both hind limbs.

The onset time of sensory blockade: Time between administration of the drug and the start of sensory blockade.

Duration of sensory blockade: A time interval during which the animal presented sensory blockade.

Time to maximum sensory blockade: Time between administering the drugs and reaching the maximum sensory blockade.

The onset time of motor blockade: Time that elapsed between administration of the drug and the start of motor blockade.

Duration of motor blockade: Time that the animal presented motor blockade.

### Data analysis

The normality of qualitative values was assessed using normal probability plots and the Kolmogorov-Simonov test generated with the UNIVARIATE procedure of SAS. All experimental data are expressed as mean ± standard deviation (STD). A one-way analysis of variance (ANOVA) was used to analyze the data, followed by Tukey-Kramer HSD for multiple comparisons to assess the analgesic effect of lidocaine and tramadol. Statistical analyses were performed using a commercial program (JMP, version 5.0.1a). The level of statistical significance for all tests was set at *p* ≤ 0.05.

## Data Availability

All data generated or analyzed during this study are included in this article.
